# Bose–Einstein condensation of photons in an erbium–ytterbium co-doped fiber cavity

**DOI:** 10.1038/s41467-019-08527-0

**Published:** 2019-02-14

**Authors:** Rafi Weill, Alexander Bekker, Boris Levit, Baruch Fischer

**Affiliations:** The Andrew & Erna Viterbi Faculty of Electrical Engineering, Technion, 32000 Haifa, Israel

## Abstract

Bose–Einstein condensation (BEC) is a special many-boson phenomenon that was observed in atomic particles at ultra-low temperatures. Later, BEC was also shown for non-atomic bosons, such as photons. Those experiments were usually done in micron-size cavities, where the power (particle number) was varied, and not the temperature, until condensation was reached. Here we demonstrate BEC of photons in a few-meters-long one-dimensional (1D) erbium–ytterbium co-doped fiber cavity at, below and above room temperature, between 100 K and 415 K. The experiments were done at about the 1550 nm wavelength regime having a few to tens of μW intra-cavity light power (10^7^−10^8^ photons). By varying the power and also the temperature, we found linear dependence of the condensation on power for various temperatures and of the critical power (for condensation) on temperature. These findings agree, functionally and quantitatively, with the theoretical BEC prediction without any adjustable parameter.

## Abstract (web summary)

Bose–Einstein condensates were demonstrated at low temperatures, mostly with ultracold bosonic atoms. Here, the authors demonstrate BEC of photons in a standard 1D erbium–ytterbium co-doped fiber cavity at, below and above room temperature.

## Introduction

After the observation of atoms Bose–Einstein condensation (BEC) at ultra-low temperatures^1–4^, there were reports on BEC with non-atomic bosons, such as photons^5,6^, exciton-polaritons, and magnons^7–13^. They were usually done in micron-sized cavities where BEC was shown by varying the power (particle number) and not the temperature as it is usually done in the atomic boson cases. For example, the pioneering experiment on photon BEC was done in a relatively simple system of a dye-filled microcavity at a room temperature^5^. These works initiated theoretical studies, discussions, and questions about the nature of photon BEC in optical cavities and its relation to lasers^8–19^. They discussed the main differences between a low loss quantum-statistics regime that can yield thermal equilibrium and BEC, and a higher losses regime of classical lasers. There were also questions regarding a lack of thermal equilibrium in some of those systems that still yielded condensation similar to BEC^9,10,19^, and also discussions about the relation to classical laser condensation^20,21^.

Photons in optical cavities and lasers that have losses were not thought to be in thermal equilibrium or show BEC. However, our recent demonstration of thermal equilibrium of a photon gas in standard erbium-doped fibers (EDF)^22^ opens the way to BEC in these simple fibers at a room temperature. We demonstrated thermalization and Bose–Einstein (BE) spectral distribution of photons in EDF in a broad spectral range up to ~200 nm at the 1550 nm wavelength regime^22^.

In this work, we experimentally observed BEC in a one-dimensional (1D) photon gas, in a standard, meters long, double-clad Er/Yb co-doped fiber (EYDF) cavity. Double-clad EYDFs that are commonly used as amplifiers for communication at the 1550 nm wavelength regime give similar thermalization behavior to what we obtained in EDF^22^, as we show below, but allow relatively uniform pumping along the fiber. Double-clad EYDFs have several advantages compared to the core EDF pumping: They give a relatively uniform upper levels population that stays in our experiments below an overall inversion along the whole fiber^22^. The uniform pumping also eliminates hysteresis and bistability^22,23^. We give in the Supplementary Notes 1 and 2 more details on the rate equations model and figures that show the differences between the cases of core and cladding pumping. We added in the cavity an important ingredient needed for BEC of a cutoff frequency, the ground state, by using an optical low-wavelength-pass filter. We discuss below its function as a cutoff. Important parts of our work that support the BEC observation besides the spectral measurements below and above condensation, the BE spectra and the thermalization, are the temperature-dependent measurements that show a close to linear dependence of the condensation light power and of the critical power needed for condensation. It follows the BEC theory for a photon gas in 1D with a linear dispersion depending only on basic quantities, the Boltzmann constant *k*_B_, and the cutoff frequency *ν*_0_, without any adjustable parameter, as shown below.

## Results

### The requirements for BEC in an EYDF cavity

The following conditions are required for obtaining BEC in fibers: (1) Thermal equilibrium that we already showed in EDF cavities, and it is similar for EYDF, as we show below. (2) Adequate light-modes density of states (DOS) in a 1D cavity, as discussed below. (3) Low loss rates of photons in the cavity that distinguishes the quantum BE-BEC regime from lasers^15,18^, fulfilled in our EYDF cavities, despite the relatively low finesse (~18) and the strong spontaneous emission loss in the fiber (~98% is lost)^22^. The low loss rate guarantees many absorption-emission cycles needed for thermalization^22^. (4) Particle (photon) number conservation, achieved by pumping the EYDF gain medium, as was discussed in the dye-filled microcavity system^5,16,17^. (5) Critical power (photon density) in the cavity or critical temperature for reaching BEC phase transition. (6) A frequency cutoff to set a reference frequency (wavelength), the ground state, for the condensation. These points can be simply obtained in EDF cavities^22^, with the special additions in this work of close to uniform pumping in the double-clad EYDF with a lack of an overall inversion along the whole fiber^22^, and a cutoff filter that with a suitable DOS and thermalization enabled the observation of BEC. We give below more elaboration on the DOS, the light power, or photon density needed for BEC in the framework of the standard BEC formalism for a photon gas in 1D. We also add in the Methods part a description of the experimental system and an elaboration on the cutoff filter.

### The density of light mode states (DOS)

The DOS generally depends on the dimension *d*, the dispersion relation, and the potential well^4^. Our fiber cavity is a 1D system, with no potential well, usually with a linear dispersion, compared to the dye-filled microcavity which is a two-dimensional (2D) transversal laser mode system with a quadratic dispersion relation for the transversal wave-vector components. For obtaining BEC, the microcavity system needed a potential trap, which was provided by two broad cavity mirrors^5,6,14,15^. It is similar to massive particle BEC that without a trap requires a dimension^4^ *d* > 2. In our case, the usual photon dispersion relation is linear, *ω*/*k* = *c*/*n*, where *ν* = *ω*/2*π* is the frequency, *k* the wavenumber, and *n* the refractive index. As we see below, a large 1D photon BEC system requires nonlinear dispersion near the cutoff, but a linear one can still yield BEC in a finite system, as the case of the present experiment. To follow the BEC formalism, we assume at some frequency region nonlinear dispersion with an exponent *η*: *ω* = *ω*_0_ + (*c*/*n*)*k*_*a*_ (*δk*/*k*_*a*_)^*η*^ or in energy terms measured from a cutoff *ε*_0_ = ℏ*ω*_0_: *ε* = ℏ(*ω* − *ω*_0_) = (ℏ*c*/*n*)*k*_*a*_ (*δk*/*k*_*a*_)^*η*^ = *b*(*δk*/*k*_*a*_)^*η*^, where ℏ = *h*/2*π* is the reduced Planck constant, *b* = (ℏ*c*/*n*)*k*_*a*_, *k*_*a*_ is a constant. We note that it is different from the usual dispersion expansion to a power series around a point with a first linear term and a second quadratic term (the GVD—group velocity dispersion). Usually, as the case in our experiments, *η* = 1. We also note that in our case, the dispersion refers to the whole cavity (and not only to the fiber), and it can be effectively modified, for example, by complex gratings. We calculate the light-modes DOS, *ρ*, for the above dispersion with an exponent *η*, here for 1D cavity with a large length *l* without a potential well^4,20–22^. $\rho (\varepsilon ) = (l/2\pi ){\int} {{\mathrm {d}}k\,\delta (\varepsilon - b(\delta k/k_a)^\eta )} = \tilde b\varepsilon ^{(1/\eta ) - 1}$, where $\tilde b = k_{a}l/(2\pi \eta b^{1/\eta })$. (In a more general case for a dimension *d*: *ρ*(*ε*)∝*ε*^(*d*/*η*)−1^.)

### The BEC transition

In thermal equilibrium, the BE distribution for the photon number is $N(\omega ) = \rho (\omega )/\left( {e^{\beta \left[ {\hbar (\omega - \omega _0) - \mu } \right]} - 1} \right)$, where *β* = 1/(*k*_B_*T*), *k*_B_ is the Boltzmann constant, *T* the temperature, and *μ* the chemical potential. BEC transition with a certain temperature *T* occurs upon having a critical photon number *N*_c_ in a cavity or at a transition temperature *T*_c_ for a certain particle number *N* or power (in the large cavity limit, with *μ* = 0 at BEC):

1 $$	N_{\mathrm{c}} = \mathop {\int}\limits_0^\infty {\rho (\varepsilon ){\mathrm{d}}\varepsilon /\left( {e^{\beta \varepsilon } - 1} \right)} = \tilde b \varGamma ({1/\eta})\zeta ({1/\eta})\left( {k_{\mathrm{B}}T} \right)^{1/\eta},\\ 	T_{\mathrm{c}} = \left({N/\tilde b\varGamma ({1/\eta})\zeta({1/\eta}}) \right)^\eta /k_{\mathrm{B}},$$

where *Γ*(x) and *ζ*(x) are the gamma and the Riemann zeta functions. Increasing the photon number *N* (or the light power) beyond *N*_c_ or decreasing the temperature below *T*_c_ solely populates the ground state (*ω* = *ω*_0_): $N_{\mathbf{0}} = N - N_{\mathbf{c}} = N - \tilde b\varGamma (1/\eta)\zeta (1/\eta)\left( {k_{\mathbf{B}}T} \right)^{1/\eta }$. Condensation is possible when the integral in Eq. 1 for *N*_c_ converges, requiring that the exponent of *ρ*(*ε*) is positive: (1/*η*)−1 > 0 or *η* < 1 (*η* > 0). (More generally for a dimension *d*: *N*_c_ ∝ (*k*_B_*T*)^*d*/*η*^, and the condition for BEC is (*d*/*η*)−1 > 0, or *η* < *d* (*η* > 0).) This means that the usual linear dispersion (*η* = 1) in a 1D system is at the boundary for BEC with a linear *T* decrease and a logarithmic divergence at *ε* = 0 of *N*_c_ as *ζ*(1/*η* → 1)→∞. However, it can yield BEC in a finite system^3^ with a cavity length *l* where the mode spacing scales as Δ*ω* = 2*πc*/*nl*. Then, the integral starts at *ε* = *ε*_1_ = ℏ*ω*_1_ = 2*π*ℏ*c*/*nl* instead of *ε* = 0, and Eq. 1 gives:

2 $$\begin{array}{ccccc}\\ N_{\mathrm{c}} \approx & \frac{{k_{\mathrm{B}}T}}{{\hbar \Delta \omega }}\ln \left( {\frac{{k_{\mathrm{B}}T}}{{\hbar \Delta \omega }}} \right) = (nl\,k_{\mathrm{B}}T/hc)\ln \left( {nlk_{\mathrm{B}}T/hc} \right) = x\,\ln \,x,\\ \\ N_0 = & N - N_{\mathrm{c}} \approx N - \left( {nl\,k_{\mathrm{B}}T/hc} \right)\ln \left( {nlk_{\mathrm{B}}T/hc} \right) = N - x\,\ln\,x,\\ \end{array}$$

where $x = nl\,k_{\mathop{\rm B}\nolimits} T/hc$, and that with a numerical insertion of the constant values become *x* = 69.46*nlT* (*lT* in meter-Kelvin units). The divergence in the integral at *ε* = 0 in Eq. 1 is removed where *ζ*(1) = ∞ is replaced by $\ln (nlk_{\mathrm{B}}T/hc)$. This condition is similar to that given for a finite 1D atomic system in harmonic potential^3^.

There can be various ways to obtain nonlinear dispersion with *η* < 1 (of the whole cavity and not necessarily of the fiber only), such as by a use of complex gratings. In this work, however, we used the fibers without any further addition to engineer the dispersion. It means that we had a linear or close to linear dispersion (*η* = 1), but a finite system, and show BEC with it, as discussed above. We note that in our experiments we obtained powers in the cavity that relate to the photon number values in Eqs. 1 and 2, by *P* = *N*ℏ*ω*_0_/*t*_rt_. Therefore, the threshold overall power is *P*_c_ = *N*_c_ℏ*ω*_0_/*t*_rt_, and the condensation power is *p*_0_ = *N*_0_ℏ*ω*_0_/*t*_rt_.

### Numerical examples for the critical photon density (power)

We estimate the critical photon density or the power in the cavity needed for BEC at *T* = 300 K. We can see from the numerical examples below that a very small and even no deviation from a linear dispersion is sufficient for obtaining BEC at moderate powers (μW–mW regime). We first use Eq. 1 for the large system (large *l*) limit which has one parameter $k_{\mathrm{a}}^{1 - 1/\eta }$ with a relatively small effect for *η* ≈ 1. For *η* = 0.9, we obtain *N*_c_/*l* ≈ 2 × 10^5^ m^−1^ or a power of *P*_c_ ≈ 5 μW (independent of length). For *η* = 0.999: *N*_c_/*l* ≈ 2.7 × 10^7^ m^−1^ and *P*_c_ ≈ 0.7 mW. As noted above, regular linear dispersion where *η* = 1, the case in our experiment, can yield BEC in finite systems. According to Eq. 2 for *l* = 27 m (the overall active EYDF fiber part was 10 m), *n* = 1.444 and *T* = 296 K, we obtain *N*_c_ ≈ 1.1 × 10^7^and *P*_c_ ≈ 9 μW, that is very close to the experimental value as can be seen below.

### Experimental observation of photon BEC in a fiber cavity

**Fig. 1:**
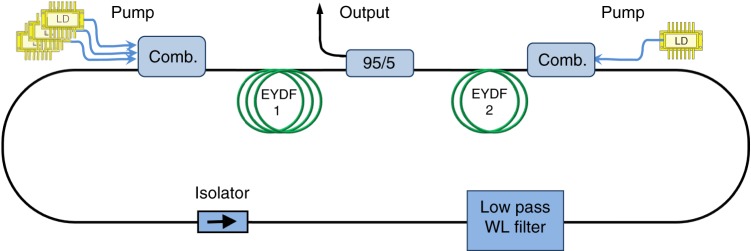
Schematic of the basic fiber cavity system. The cavity contains two gain sections of double-clad Er/Yb co-doped fiber 1 (EYDF1) and EYDF2 with lengths of 9 and 1 m, respectively, two sets of laser diodes (LDs) pumping the EYDF through combiners from two sides to make the pump more uniform, a low-pass wavelength (WL) filter with a cutoff at *λ*_0_ = 1568 nm, and an isolator

**Fig. 2:**
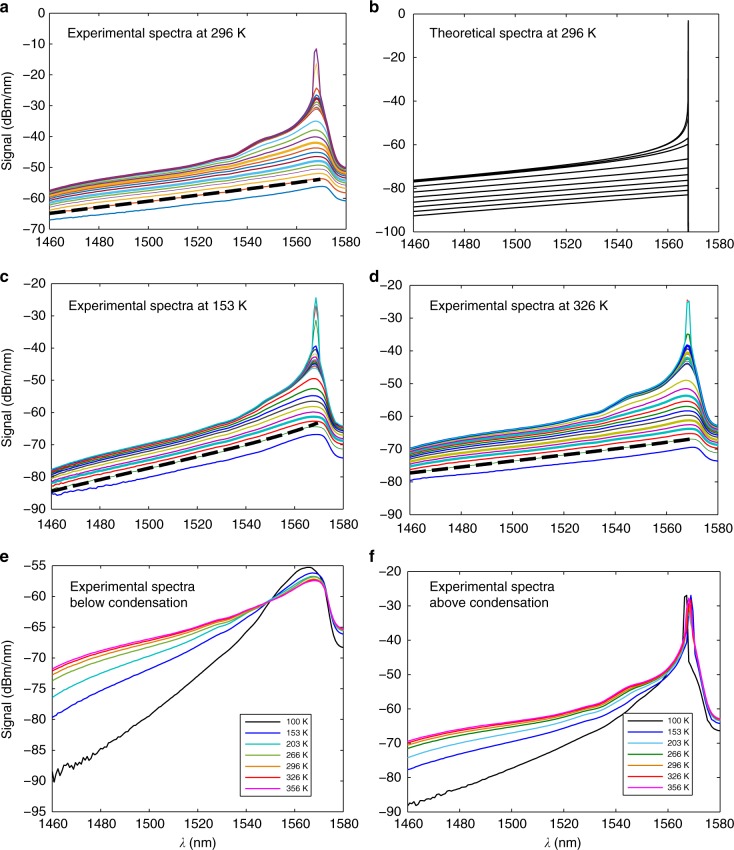
Experimental and theoretical spectra below and above condensation. They are given for different overall powers (photon density) *P* and temperatures *T*. The slopes of the almost straight lines in the spectra (*λ* dependence of *hν*/*k*_B_*T*) fit the theoretical thermal Bose–Einstein (BE) distribution slope for each temperature shown by the dashed lines. **a** Experimental spectra at a room temperature (296 K) for various intra-cavity power *P* values. **b** Theoretical BE distribution at a room temperature. **c** Experimental spectra at 153 K. **d** Experimental spectra at 326 K. The different power *P* values for the spectra in **a**, **c**, **d** are obtained by increasing the pumping from 0 to 80 μW. **e**, **f** show the spectra for various temperatures, each with a fixed power, below and above condensation (*P* = 1.4 and 80 μW, respectively). We emphasize that the slopes match the various temperatures following the corresponding BE spectra

The experimental system of the fiber cavity is shown in Fig. 1 and it is described in the Methods section. The temperature dependence of BEC is the most important part of the present work. It allows a direct verification of the condensation as the temperature is varied, as it is usually done with atomic boson BEC, but has not been done yet in non-atomic or photon systems. The first results are the spectra in Fig. 2. They are shown at, below, and above the BEC formation for different power levels and temperatures. We can see in all of them the broad BE distribution with the almost straight line (*λ* and not *ν* dependence) in a semi-log scale in a range of ~100 nm prior to the BEC buildup, which fit the temperature-dependent BE distribution. We stress what we point out in the Supplementary Note 2 and in ref. ^22^ that the thermalization drastically transforms the spectrum to a broad BE distribution and the initial spectrum is less important. Above a certain pumping and intra-cavity critical light power *P*_c_ we have a buildup of the condensation near the cutoff, while keeping the broad spectra beside the condensation almost unchanged, in accordance with the BEC theory. The condensation is near the wavelength cutoff at *λ*_0_ = 1568 nm that corresponds to a frequency *ν*_0_ = *ω*_0_/2*π*. The theoretical spectra (Fig. 2b and the dashed lines in Fig. 2a, c, d, obtained in a similar way to our work in ref. ^22^), with a cutoff filter that falls to zero above 1568 nm, show a very good agreement with the experimental graphs in Fig. 2, including an excellent fit to the BE distribution for the various temperatures. Figures 2e, f show the spectra dependence on the temperature for fixed powers, below and above the condensation formation, at *P* = 1.4  and 80 μW, respectively. We note that there is a slight deviation at ~1550 nm from a smooth BE spectrum, but the power there is ~(10–30) dB lower than the power in the condensation mode, and therefore it has a negligible effect on the coherence properties of the single mode light. We think it is because the cross-sections do not satisfy the McCumber (or Kennard-Stepanov) relation^22–28^ to infinite precision, and there are some points where the ratio is not exactly $\exp (\hbar \omega /k_{\mathrm{B}}T)$, as can be seen in Supplementary Fig. 5. Near 1550 nm the absorption/emission cross-sections are high and therefore any slight deviation will cause a slight change of the thermal spectra. We also show in the Supplementary Note 2 that without a filter, the spectral range of the thermalization is extended for all powers, with no oscillation at 1568 nm, to wavelengths above 1600 nm, up to the point where the emission-absorption region ends.

**Fig. 3:**
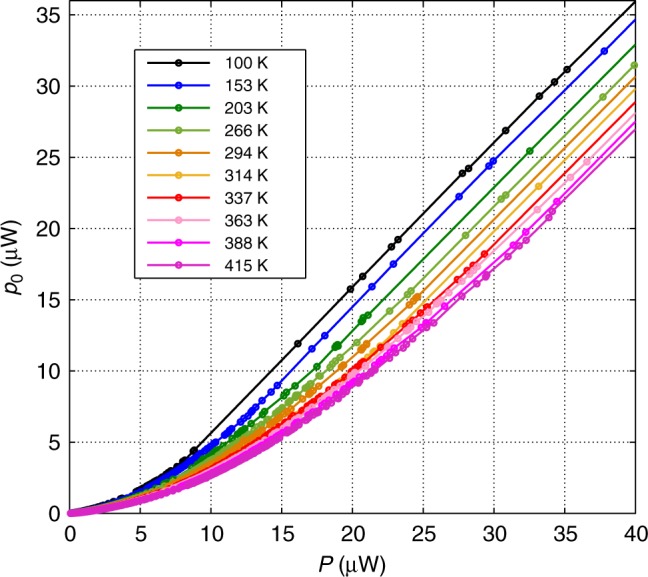
Experimental condensation *P* dependence for various temperatures. The condensation is given by the light concentration near the cutoff wavelength. Beside the initial slow grow part near zero *P*, the graphs are almost straight lines showing a linear *P* dependence with a similar slope of 1, in accordance with the theoretical analysis

**Fig. 4:**
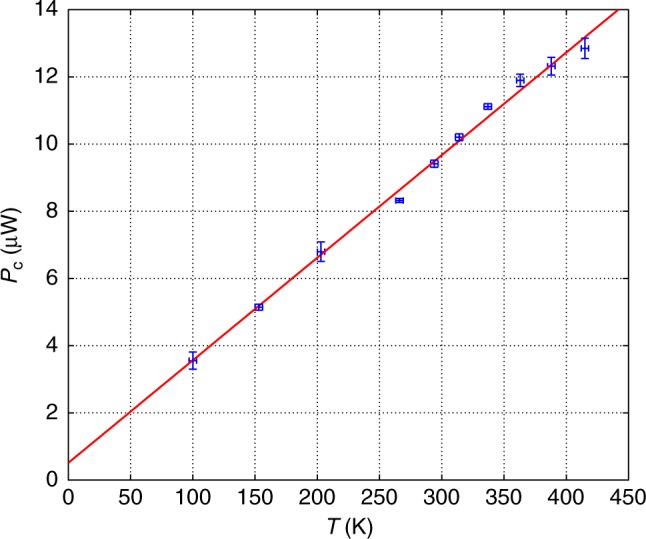
Threshold power *P*_c_ dependence on temperature *T*. It is obtained by the intersection with the *P-*axis of the linear parts fit in Fig. 3. The graph gives an almost linear dependence in accordance with the theoretical result of *P* = *aT*. The slope in the experiment is (0.031 ± 0.003) μW/K, close to the theoretical value *a* ≈ 0.034 μW/K without any adjustable parameter. The error bars result from uncertainty in the temperature measurements of the fiber core, as well as s.d. in the fitting of the linear parts of the graphs in Fig. 3

Figures 3 and 4 show experimental results where the temperature was varied in the range of (100–415) K. We can see in Fig. 3 the condensation power *p*_0_ dependence on the intra-cavity power *P* for various temperatures. The condensation is given by the light concentration near the cutoff wavelength. We used for the *p*_0_ measurement a spectral width of 2 nm since smaller values gave noisy results near the cutoff. The initial small nonzero growth of the graphs near zero *P*, and the non-sharp transition before they become straight lines with a slope 1, is common in phase transitions of finite system and matches the calculations. The linear *P* dependence is in accordance with the theoretical analysis given in Eq. 2 for an exponent *η* = 1 of the dispersion (*δω* ∝ *δk*^*η*^) near the cutoff frequency *ν*_0_ = *ω*_0_/2*π* with a conversion of the photon numbers to power by a factor ℏ*ω*_0_/*t*_rt_, where *t*_rt_ = *l*/(*c*/*n*) is the round-trip time of light in the cavity. It gives *p*_0_ ≈ *P* − (ℏ*ω*_0_/*t*_rt_)*x* ln*x* ≈ *P* − *aT* with a constant slope *a* ≈ *ν*_0_*k*_B _ln *x* ≈ 0.034 μW/K, where *x* = *nlk*_B_*T*/*hc*. We took an average value for the factor ln *x* ≈ 13, which does not vary much in the relevant temperature range. We emphasize that the slope *a* depends only on basic quantities *k*_B_ and *ν*_0_ and not on fiber cavity or doping parameters, except for a weak logarithmic dependence on the cavity length *l* in ln *x* = ln (*nlk*_B_*T/hc*). Therefore, we have an approximately linear *p*_0_ dependences on *P* and *T* (the latter with a slope *a*). The critical power, obtained where *p*_0_ = 0, is given by *P*_c_ ≈ *aT*. Figure 4 verifies this result experimentally. The threshold power *P*_c_ dependence on temperature *T* is obtained by the intersection of the extended linear lines in Fig. 3 with the *P*-axis. The graph in Fig. 4 gives an almost linear dependence with a slope (0.031 ± 0.003) μW/K, close to the theoretical value of *a* ≈ 0.034 μW/K without any adjustable parameter. *P*_c_(*T* = 0) in the experiment is slightly above zero, probably because of cavity losses by the coupler and other elements. The experimental results agreement with the theory is excellent. For example, the experimental value for the critical power in the cavity for condensation given in Fig. 4 for a temperature *T* = 296 K is *P*_c_ ≈ 9.5 μW. It is very close to the theoretical prediction of *N*_c_ ≈ 1.1×10^7^and *P*_c_ ≈ 9 μW for *T* = 296 K, as was shown above (calculated from Eq. 2 for *l* = 27 m). We can also deduce from Fig. 3 the condensation power *p*_0_ dependence on temperature *T* for various constant powers, obtained by the intersections of the lines in Fig. 3 with any vertical line (constant *P*) that gives a *p*_0_ ≈ *P* − *aT* dependence as the theory predicts. Extrapolation to the *T*-axis gives the BEC transition temperatures (*p*_0_ = 0) that match the theoretical result of *T*_c_ = *P*/*a*. For example, for *P* = 14 μW, it gives a BEC transition at *T*_c_ ≈ 451 K.

## Discussion

The experimental results excellently follow the theory, also quantitatively, depending only on basic quantities (*k*_B_ and *ν*_0_) without any adjustable parameter, and thus provide a strong basis for the photon BEC observation in our fiber system. Therefore, besides the low loss-rate quantum regime of our fiber cavity that distinguishes it from the laser cases, the BEC observation is based on the thermalization with the broad BE spectra below and above condensation for various temperatures, and the temperature-dependent measurements of the condensation and the critical powers, which all match the theoretical and numerical photon BEC predictions in 1D.

We add in the Supplementary Notes 1 and 2 a detailed description with figures that show the matching of the experimental and theoretical results and the differences between core and cladding pumping cases, based on the rate equations with the parameters given in Supplementary Table 1. We also show there that without a filter the thermalization range in the theoretical and experimental spectra is extended to wavelengths above 1600 nm, up to the point where the emission-absorption region ends.

## Methods

### The experimental system

We used a ring cavity, schematically shown in Fig. 1, with a gain medium provided by two sections of double-clad EYDFs with lengths of 9 and 1 m and an overall cavity length of *l* ≈ 27 m. The active EYDF had a relatively high erbium concentration (having a peak absorption of 0.89 dB/m near 915 nm and 39.5 dB/m near 1535 nm). Double-clad EYDFs can be pumped more uniformly than single clad EDFs, and therefore enables a relatively uniform overall upper levels population and the elimination of hysteresis^22^, but otherwise shows similar properties with similar absorption-emission spectra and their ratio behavior that follows the McCumber or Kennard-Stepanov relation^22–28^. (More details can be found in Supplementary Notes 1, 2, and 3.) We used for the cutoff frequency a fiber pigtailed low loss (<0.8 dB) low-wavelength pass filter with a cutoff at *λ*_0_ ≈ 1568 nm, and a rejection ratio of ~30 dB between the pass and loss regions. The filter was placed after the first EYDF section, but before the second one that was followed by the output coupler, to allow thermalization but reduce its direct effect on the output. We also had in the cavity an isolator to allow a unidirectional light propagation (counterclockwise) with a rejection ratio of 40 dB and a loss of 1 dB. The loss was due to an output coupler of ~5%, which was placed between the two EYDFs, and from other losses in the connectors and the above-mentioned elements. The overall loss in the cavity was about 30%, but the photon cavity lifetime is *t*_c_~(10^−6^–10^−7^) s, which put the cavity in the low loss-rate quantum regime^18^. It is due to the relatively long cavity length of 27 m, although the finesse was relatively low (~18) compared to the dye-filled microcavity system^5,6,16,17^, which had a very high mirror reflectivity of (~0.999985) and very high finesse (10^4^–10^5^) but a short length of ~1.5 μm that give *t*_c_ ~ 0.3 × 10^−9^ s. Our system was pumped by laser diodes at 915 nm coupled to the fiber system at the ends of the two EYDF sections and propagate oppositely to give a relatively uniform pumping. Discussions that the system obeys the constant photon number requirement in the cavity by pumping the gain medium that compensates for the lost photons, and that it provides a grand canonical ensemble were given in former works^5,16,17^. The dispersion relation of light in fibers is close to the standard linear (*η* = 1) relation (besides a small effect of normal GVD of EYDF at the 1550 nm wavelength regime that can provide a small sublinear deviation). Therefore, we have a 1D fiber system which is at the boundary of BEC, but as we showed it can yield BEC in a finite system.

For the temperature variation, we used a heating chamber with a controlled temperature that was measured by three elements: a fiber grating with a Bragg wavelength that varied with temperature, a thermocouple, and a temperature-dependent resistor. For cooling, we used a cryostat system with liquid nitrogen in a dry dewar where the EYDF was placed in different heights to have various temperatures. We could extend the temperature range from what we have in this paper, but here we chose to stay at a safe region not to damage the fiber system.

### The cutoff filter

As mentioned above, we added in the cavity an optical low-wavelength pass filter that serves as a cutoff frequency needed for BEC. We note that its effect is different from the prohibited wavelength regimes between the longitudinal modes in a Fabry–Perot cavity used for the cutoff in the microcavity experiment^5,6^, especially when the rejection (30 dB) ratio of the filter was not infinite and the cutoff width was not sharp (≈5 nm). It works on the loss in the “suppressed” region that makes the photon cavity lifetime *t*_c_ close to zero beyond the cutoff, rather than nulling the DOS. Nevertheless, the theoretical calculation and the rate equation model give the same results for both cases^22^, as can be understood when strong losses like from Bragg reflections suppress oscillation in a cavity and in solids resulting in frequency or energy band gaps. It would be preferable to have a distribution of filters along the cavity, but in practice we used one filter that provided the needed cutoff function. The uniform pumping helps to treat the fiber cavity as a lumped system and adds justification to the discrete filter role as a cutoff.

## Supplementary information

Supplementary Information

## Data Availability

The data that support the findings of this study are available from the authors upon request.
