# Supplementary material for: Bose–Einstein condensation of photons in an erbium–ytterbium co-doped fiber cavity
Source: Nat Commun. 2019 Feb 14;10:747. doi: 10.1038/s41467-019-08527-0 (PMC6376030; doi:10.1038/s41467-019-08527-0)
Supplement: Supplementary file 1 — Supplementary Information [file 41467_2019_8527_MOESM1_ESM.pdf]

Supplementary Information

for the paper

Bose-Einstein condensation of photons in an Erbium-Ytterbium co-doped fibre cavity

Rafi Weill, Alexander Bekker, Boris Levit, and Baruch Fischer

The Andrew & Erna Viterbi Faculty of Electrical Engineering, Technion, Haifa 32000, Israel

E-mail: fischer@ee.technion.ac.il

We add here a more detailed description with figures that show the differences between core and cladding pumping, with and without cutoffs. Without a cutoff filter, the spectral range of the thermalization is extended to wavelengths above 1600 *nm*, up to the point where the emission-absorption region of Erbium doped or Erbium-Ytterbium co-doped fibers (EDF/EYDF) ends.

Supplementary Note 1: The rate equations model with numerical calculations

To understand the thermalization and the condensation processes in EDF and EYDF cavities, with and without cutoff filters, and to distinguish between these cases, we give a simple numerical model that describes the signal and pump propagation along the fibre (*z*-axis). Most features can be obtained by the following simple distributed rate equations (and some important aspects can be obtained even by a single lumped rate equation that we describe below)[1]:

$$dP(z)/dz = -\Gamma_{\mathrm{p}}\sigma_{\mathrm{p}}N_1(z) \tag{1}$$

$$dp(\nu,z)/dz = [\sigma_{21}(\nu)N_2(z) - \sigma_{12}(\nu)N_1(z)]p(\nu,z) + \kappa N_2(\mathrm{z})\sigma_{21}(\nu)\,\mathrm{g}(\nu)h\nu \tag{2}$$

$$dN_2(z)/dt = \sigma_{\mathrm{p}}\Gamma_{\mathrm{p}}N_1(z)\frac{P(z)}{h\nu_p A_{\mathrm{c}}} + \int d\nu\sigma_{12}(\nu)N_1(z)\frac{p(\nu,z)}{h\nu A_{\mathrm{c}}} - \int d\nu\sigma_{21}(\nu)N_2(z)\frac{p(\nu,z)}{h\nu A_{\mathrm{c}}} - \frac{N_2}{t_{\mathrm{sp}}} \tag{3}$$

$$N_1(z) + N_2(z) = N_{\mathrm{d}} \tag{4}$$

Supplementary equation (1) describes the pump power $P(z)$ absorption by the ground level atoms. The distinction between core and cladding pumping is given by the pump-active fibre (core signal mode) overlap parameter Γ_{p}. The starting condition is determined by the pump input value $P(z=0) = P_{\mathrm{in}}$. Supplementary equation (2) describes the propagation of the signal mode power, $p(\nu,z)$, from $z=0$ to $z=l_a$. It is governed by frequency dependent absorption, stimulated and spontaneous emission. We have for Supplementary equation (2) the following boundary condition: The output coupler, located at $z=l_a$, couples out a fraction (*1-R*) of the power and returns a fraction *R* back to $z=0$, ($p(\nu,0) = R\,p(\nu,l_a)$). Supplementary equations (3) and (4) describe the population dynamics of the ground and excited levels N_1, N_2. We use the two-level approximation that assumes very fast transition from the pump excited levels (Yb)

to the excited levels 2 (Er). We note that each of these levels is broad and comprises a band of sublevels. The parameters description and their values are listed in Supplementary Table 1.

**Parameter**	**Symbol**	**Value**	**Unit**
Position along the fibre	z	0-10	*m*
Frequency	ν	$1.852\times10^{14} - 2.069\times10^{14}$	*Hz*
Pump power	P		*W*
Signal power per frequency	$p(\nu)$		*W/Hz*
Pump/signal-core-mode overlap	Γ_p	0.005 for cladding pumping<br>1 for core pumping	-
Pump absorption cross section	σ_p	$2.58\cdot10^{-25}$	m^2
Stimulated emission cross section	σ_{21}	See Supplementary Fig. 5	m^2
Stimulated absorption cross section	σ_{12}	See Supplementary Fig. 5	m^2
Spontaneous emission coupling to fibre mode	κ	0.02	-
Density of light mode states (DOS)	$g(\nu)$	2	-
Ground levels population	N_1		m^{-3}
Excited levels population	N_2		m^{-3}
Number concentration of erbium atoms	N_d	$4.91\cdot10^{25}$	m^{-3}
Core area	A_c	$2.38\cdot10^{-11}$	m^2
Excited levels decay time	t_{sp}	10	*ms*
Active fibre (EDF/EYDF) length	l_a	10	*m*

Supplementary Table 1: The model parameters. The parameters and their values that we used for obtaining the theoretical figures from the rate equations.

Supplementary Note 2: Core vs. cladding pumped cavities, with and without a cutoff filter

The pump/signal-core-mode overlap parameter Γ_p controls the pump behavior: For core pumping (in the EDF case) Γ_p ~1, and for the cladding pumping EYDF case Γ_p is small (slightly above zero). The filter is modeled by the transfer function $F(\lambda)$) that multiplies the signal at z=9 *m* (1 *m* before the output coupler). We assume for simplicity an ideal filter: $F(\lambda \le 1568nm) = 1$ and $F(\lambda > 1568nm) = 0$.

Supplementary Fig. 1 shows the results obtained with the model for cladding and core pumped cavities with a cutoff filter. Fig.1a shows for the cladding pumping case (EYDF), thermalization spectra and then a continuous transition to condensation as the pumping power is increased. This picture fits the experimental results in the present paper. For the core pumping case, Fig. 1b shows development to thermalization spectra and a jump and abrupt transition to condensation (and hysteresis) when the pumping is increased[1,2].

To better understand the differences between the two cases we show in Supplementary Fig. 1c,d the spectra at z=1 m, close to the pump origin, and in Supplementary Fig. 1e,f the population ratio N_2 / N_d as a function of z. For the cladding pumping case the spectra at z=1 m are similar to the output spectra, and the population ratio remains almost constant, independent of z. (In the experiment we keep the pumping and the population ratio even more uniform by pumping from two sides). This means that every point in the cavity sees the same conditions, and even the lumped system rate equation that is[1]:

$$dp(\nu)/dt = \kappa N_2 B_{21}(\nu) g(\nu) h\nu + N_2 B_{21}(\nu) p(\nu) - N_1 B_{12}(\nu) p(\nu) \ , \qquad (5)$$

leads to thermalization and condensation and is well suited to the system. $B_{12}(\nu)$ and $B_{21}(\nu)$ are the absorption/emission rates, related to $\sigma_{12}(\nu)$ and $\sigma_{21}(\nu)$, where $\sigma_{12}(\nu)/\sigma_{21}(\nu) = B_{12}(\nu)/B_{21}(\nu) \propto e^{\beta h\nu}$ [1,4,5,6,7,8]. However, for the core pumped case, below threshold, the pump is mostly absorbed after a short distance from the input and therefore the excited levels population is high only near the pump input. The signal in this region shows typical spectra of erbium emission, but as it propagates in the un-pumped region it thermalizes, mainly by absorption. Above threshold, there is enough signal to maintain the excited state population at the required threshold value which is equal to the cladding pumping case. This state is therefore similar to the condensate state of the cladding pumping case.

As shown in Supplementary Fig. 1, our model also explains the tail in the spectrum below the cutoff frequency ($\lambda > 1568\,nm$). Although the filter is ideal, there is a buildup of spontaneous emission at the 1 m long fibre between the filter and the output coupler. This spontaneous emission is not affected by the cavity conditions since it takes less than one roundtrip to build it up. We believe that the spontaneous emission in one roundtrip which is unaffected by the cavity mirrors or the density of states is also the reason for the spectrum tail in the microcavity experiment[3].

Supplementary Fig. 2 shows the results obtained with the model for cladding and core pumped cavities without filters. Supplementary Fig. 2a,b shows the output power spectra for the two cases. For the cladding pumping case (Fig. 2a), the spectra show thermalization and then oscillation, (probably lasing) in multiple modes between (1600-1620) *nm*. The transition between thermalization to lasing is continuous and resembles the condensation transition, but since the cutoff mode is not well defined, and the emission and absorption cross sections at the region (1600-1620) *nm* don't agree with the McCumber (or Kennard-Stepanov) relation[1,4,5,6,7,8] there is an undefined state of oscillation. The results shown in Supplementary Fig. 2b for the core pumping case can be compared to the experimental results we reported in Supplementary Ref. 1 that show a broad thermalization region and a jump and abrupt transition to spectra with a broad thermalization region and oscillation at the band edge, above 1600 *nm*, when the pumping was increased. The spectra for the cladding pumping case can be compared to the experimental results in Supplementary Fig. 3 shown here. Without a filter there is no BEC power accumulation near the cutoff wavelength at 1568 *nm* and the thermalization region is extended to wavelengths above 1600 *nm* up to the point where the emission-absorption region ends. The experimental results show oscillation at ~1610 *nm* on top of the thermalized spectra in a large but defined band.

Cladding pumped cavity with filter **Core pumped cavity with filter**

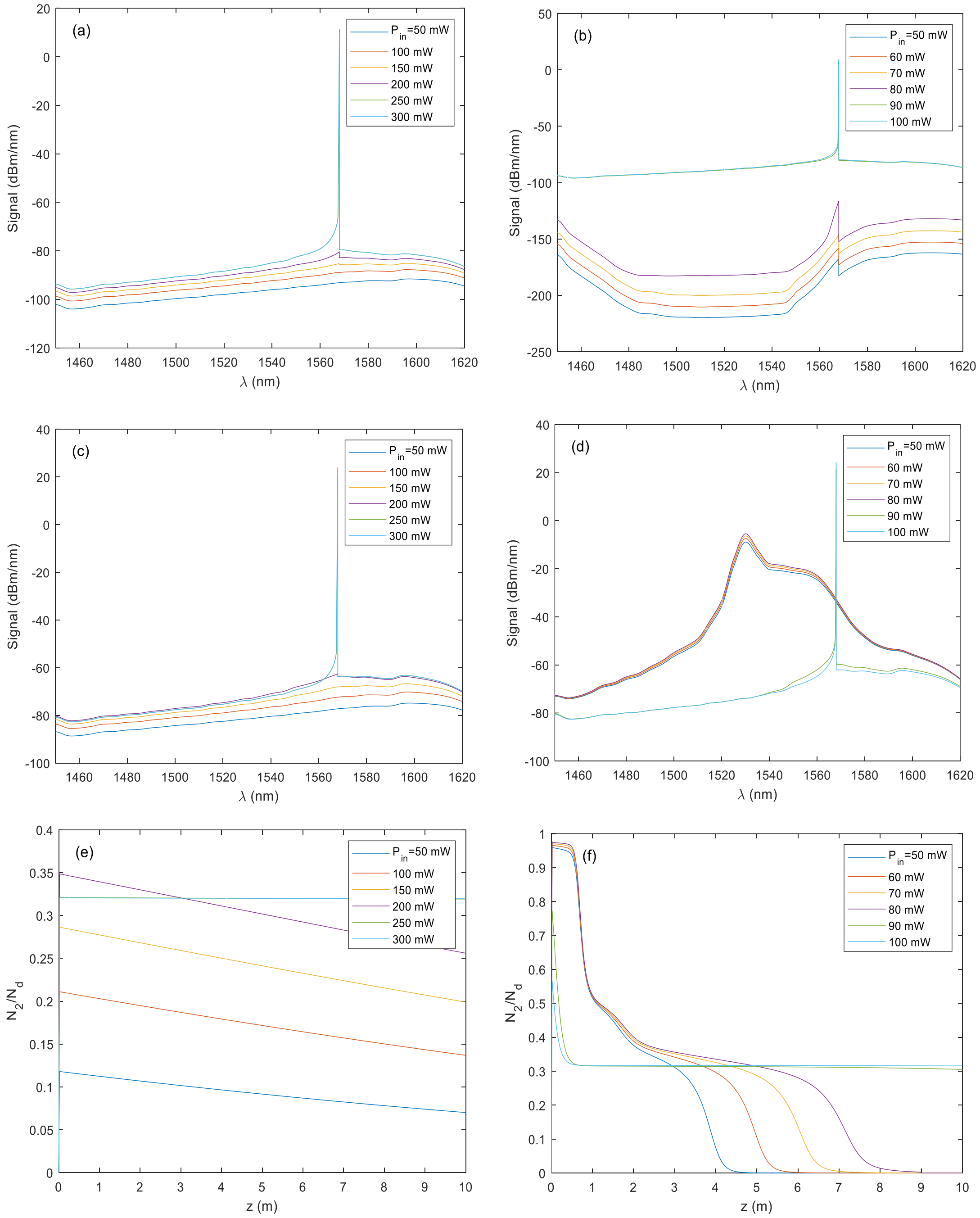

Supplementary Figure 1: Results obtained with the model for cladding and core pumped cavities with a cutoff filter. The output power spectra for (a) - cladding pumped cavity, and for (b) - core pumped cavity. The spectra inside the cavity at z=1 m for (c) - cladding pumped cavity, and for (d) - core pumped cavity. The population ratio of the excited level N_2/N_d as a function of z for (e) - cladding pumped cavity, and for (f) - core pumped cavity.

Cladding pumped cavity without filter **Core pumped cavity without filter**

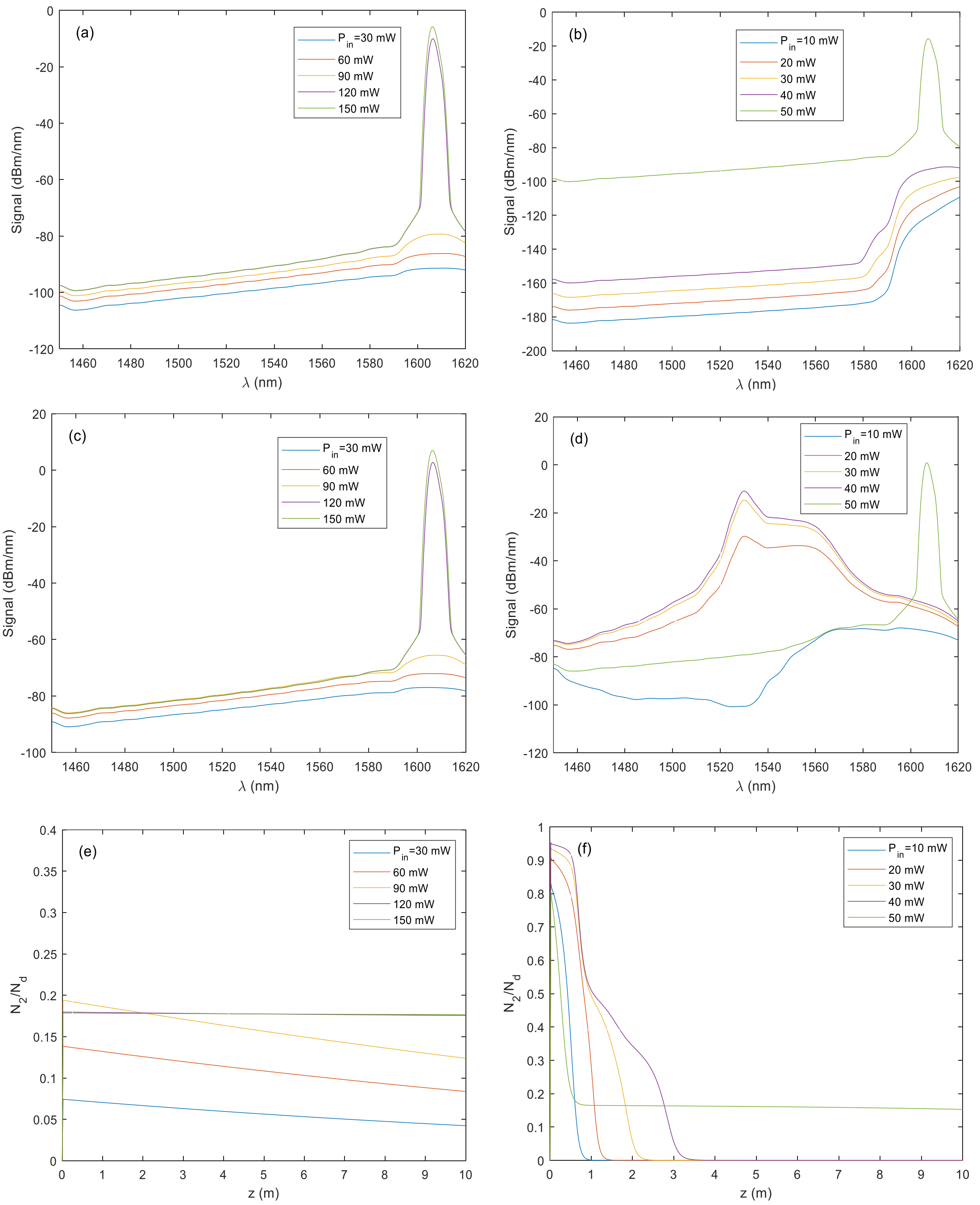

Supplementary Figure 2: Results obtained with the model for cladding and core pumped cavities without cutoff filters. The output power spectra for (a) - cladding pumped cavity, and for (b) - core pumped cavity. The spectra inside the cavity at position z=1 m for (c) - cladding pumped cavity, and for (d) - core pumped cavity. The population ratio of the excited level N_2/N_d as a function of z for (e) - cladding pumped cavity, and for (f) - core pumped cavity.

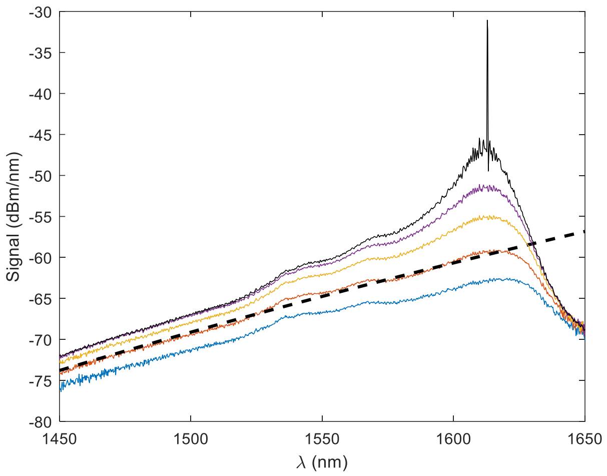

Supplementary Figure 3: Experimental spectra measurements at 296 *K* of a cladding pumped cavity without a filter. The dashed line shows the theoretical slope of the BE distribution for this temperature.

Supplementary Note 3: Additional data on the emission and absorption cross sections and the amplified spontaneous emission spectra

We give here additional data supporting the claims for thermalization and BEC. If the cavity has a low loss-rate that supports many absorption-emission cycles the signal spectra will not be dominated by the spectral shape of the emission and absorption cross sections. Instead, it depends only on their ratio, which is temperature dependent. To show that the spectra we measured in the cavity are indeed very different from the usual gain spectra, we experimentally measured the amplified spontaneous emission (ASE) spectra. Supplementary Fig. 4 shows experimental ASE in a 1 *m* long open double-clad EYDF that we used in the present paper for different pump power levels at three temperature values 100 *K*, 296 *K* and 413 *K* that cover the range that we used. They show a small effect of the temperature in the ranges we used. We emphasize that the thermalizaion drastically transforms the whole spectrum to a very broad BE distribution (where the initial spectrum is less important to this change), and the generation of BEC near the cutoff when the power is high enough, as we see in this paper and Supplementary Ref. 1 (on thermalization). Since there is a difference between spontaneous emission that is usually taken for short distances and ASE (that is here taken for 1 *m* propagation in the fibre) we used for our modeling the spectra given in Supplementary Fig. 5.

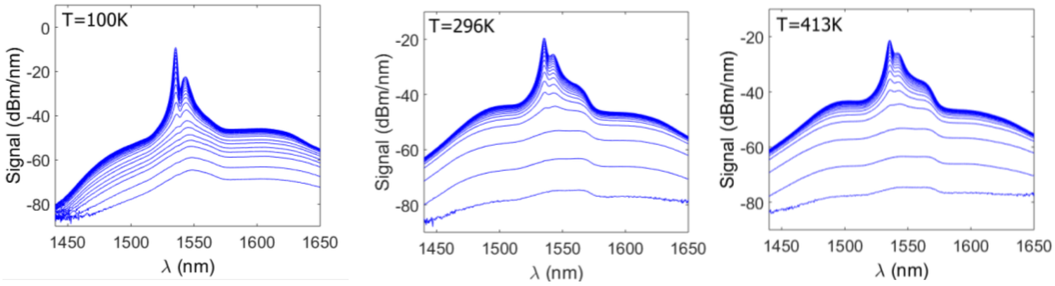

Supplementary Figure 4: Experimental spectra of ASE in an open 1 *m* EYDF. They are given for three temperatures and different pumping power levels (that are not specified since only a small part of it is absorbed in the fibre in the cladding pumping case.) The temperatures, 100 *K*, 296 *K* and 413 *K*, cover the range that we used (room, lowest and highest values). They show that there is a relatively small effect of the temperature on the ASE spectra in the temperature range that we used. We emphasize that upon a long enough propagation the thermalizaion drastically transforms the whole spectrum to a BE distribution and eventually to BEC.

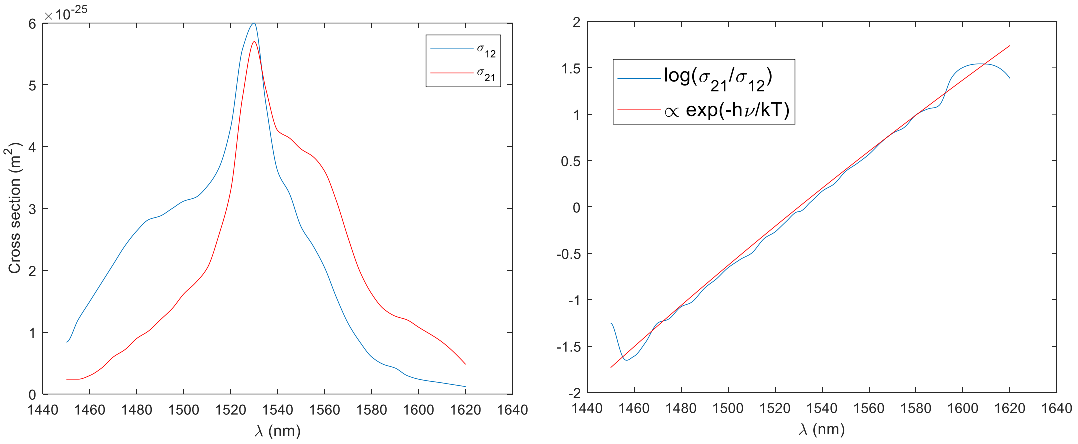

Supplementary Figure 5: Stimulated emission and absorption cross sections of the erbium-doped fibre that we used in our simulation. The right figure shows the ratio of the two cross sections showing that they follow the McCumber (or Kennard-Stepanov) relation[1,4--8] at most of the wavelength band.

Supplementary References

1. Weill, R., Bekker, A., Levit, B., Zhurahov, M. & Fischer, B. Thermalization of one-dimensional photon gas and thermal lasers in erbium-doped fibers. *Opt. Express* **25**, 18963-73 (2017).
2. Zhurahov, M., Weill, R., Bekker, A., Levit, B., & Fischer, B. Bistability in a ring erbium-doped fiber laser due to ejected backward amplified spontaneous emission. *Opt. Express* **25**, 32451-56 (2017).
3. Klaers, J., Schmitt, J., Vewinger, F. & Weitz, M. Bose-Einstein condensation of photons in an optical microcavity. *Nature* **468**, 545-548 (2010).
4. McCumber, D. E. Theory of phonon terminated optical masers. *Phys. Rev.* **134**, A299 (1964).
5. R. M. Martin, R. M. & Quimby, R.S. Experimental evidence of the validity of the McCumber theory relating emission and absorption for rare-earth glasses. *J. Opt. Soc. Am. B*, **23**, 1770-1775 (2006).
6. Desurvire, E. *Erbium-doped fiber amplifiers*, Wiley Publication (1994).
7. Kennard, E.H. On the Interaction of Radiation with Matter and on Fluorescent Exciting Power. *Phys. Rev*. **28**, 672-683 (1926).
8. Stepanov, B.I. Universal relation between the absorption and luminescence spectra of complex molecules. *Dokl. Akad. Nauk SSR* **112**, 839-841 (1957).
